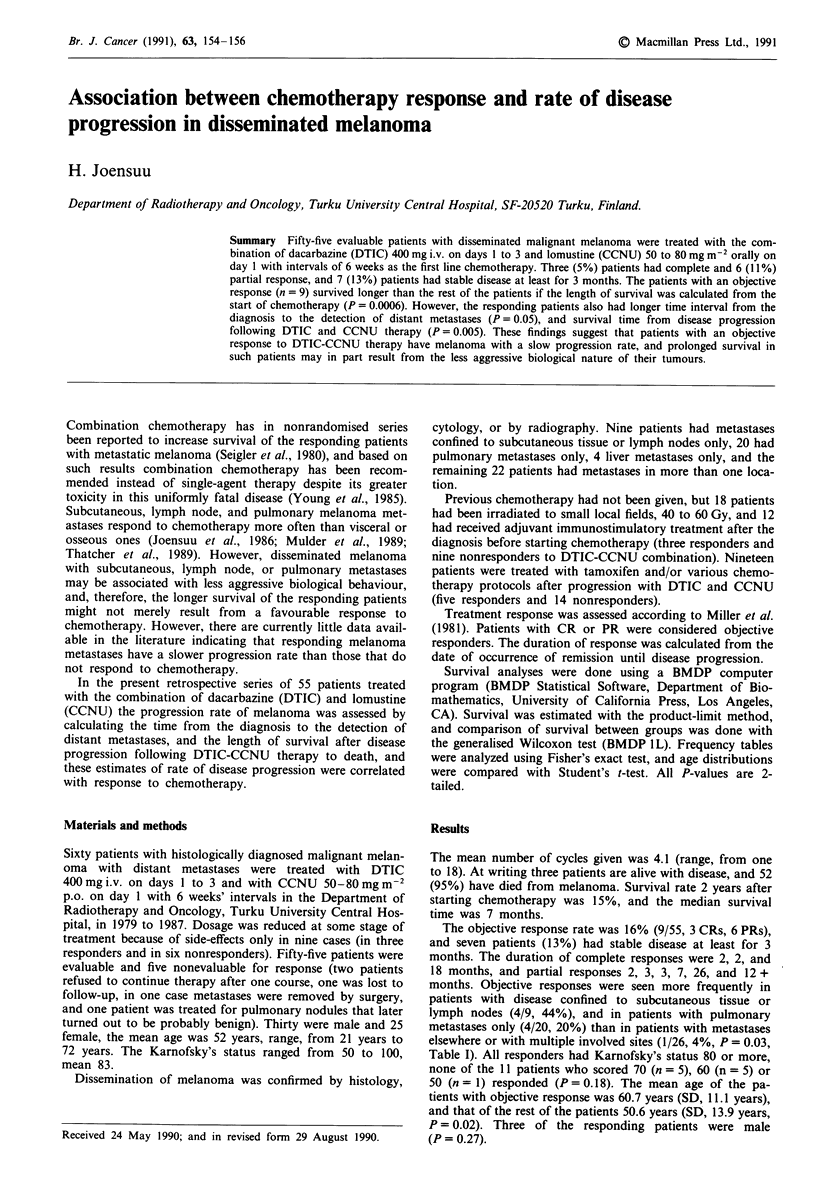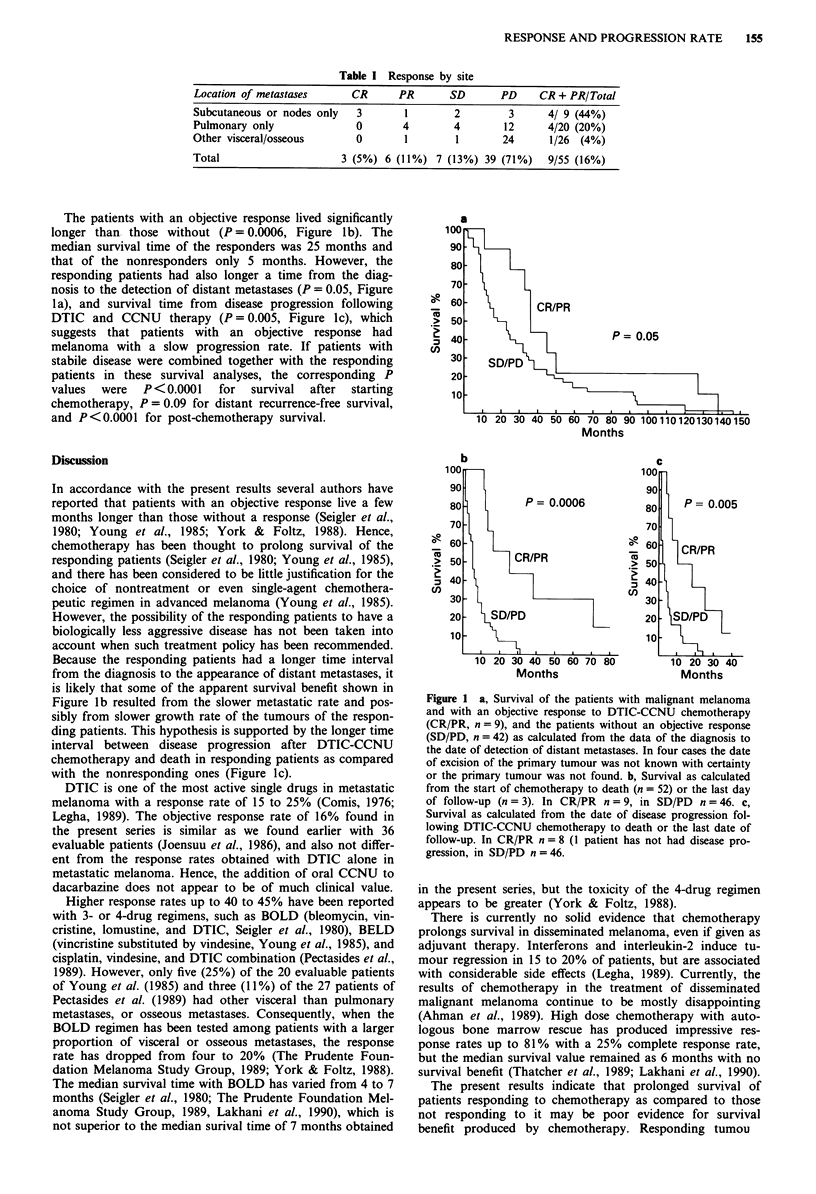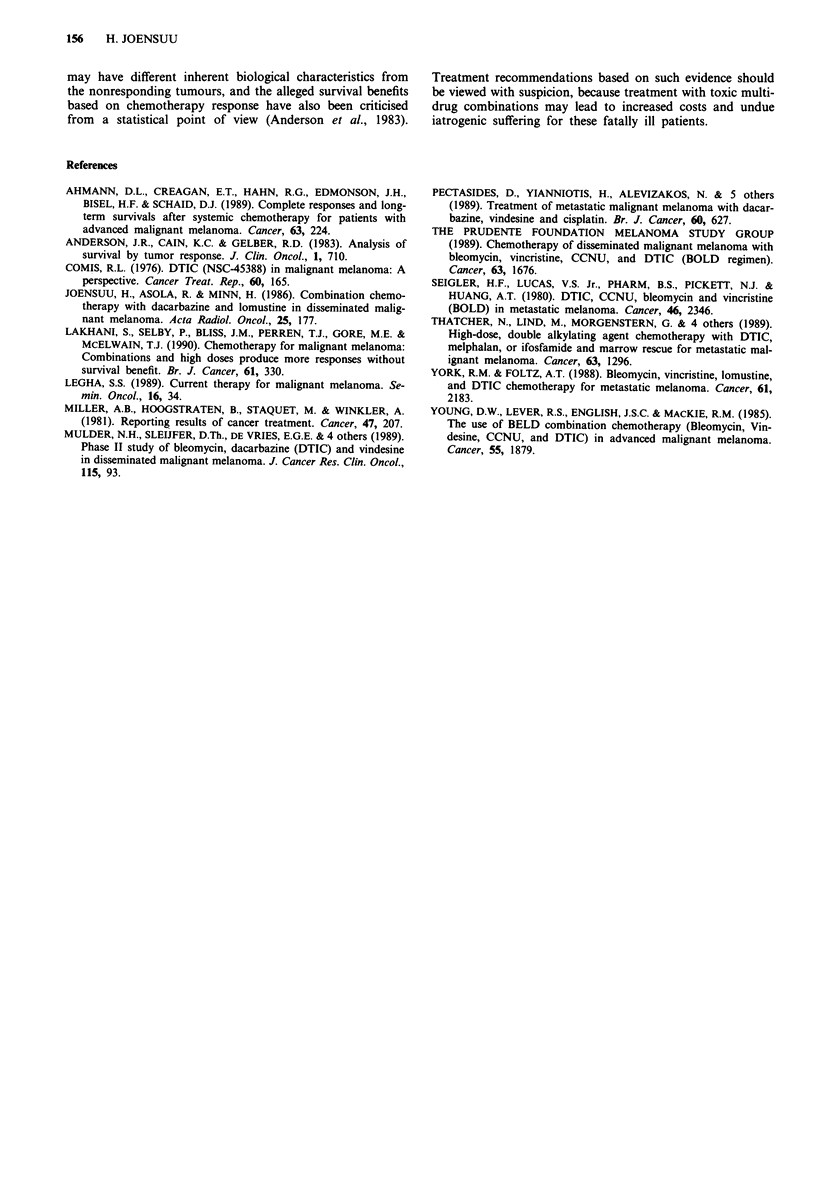# Association between chemotherapy response and rate of disease progression in disseminated melanoma.

**DOI:** 10.1038/bjc.1991.32

**Published:** 1991-01

**Authors:** H. Joensuu

**Affiliations:** Department of Radiotherapy and Oncology, Turku University Central Hospital, Finland.

## Abstract

Fifty-five evaluable patients with disseminated malignant melanoma were treated with the combination of dacarbazine (DTIC) 400 mg i.v. on days 1 to 3 and lomustine (CCNU) 50 to 80 mg m-2 orally on day 1 with intervals of 6 weeks as the first line chemotherapy. Three (5%) patients had complete and 6 (11%) partial response, and 7 (13%) patients had stable disease at least for 3 months. The patients with an objective response (n = 9) survived longer than the rest of the patients if the length of survival was calculated from the start of chemotherapy (P = 0.0006). However, the responding patients also had longer time interval from the diagnosis to the detection of distant metastases (P = 0.05), and survival time from disease progression following DTIC and CCNU therapy (P = 0.005). These findings suggest that patients with an objective response to DTIC-CCNU therapy have melanoma with a slow progression rate, and prolonged survival in such patients may in part result from the less aggressive biological nature of their tumours.


Br. J. Cancer (1991), 63, 154–156 © Macmillan Press Ltd., 1991

### Association between chemotherapy response and rate of disease progression in disseminated melanoma

H. Joensuu

*Department of Radiotherapy and Oncology, Turku University Central Hospital, SF-20520 Turku, Finland.*

**Summary** Fifty-five evaluable patients with disseminated malignant melanoma were treated with the combination of dacarbazine (DTIC) 400 mg i.v. on days 1 to 3 and lomustine (CCNU) 50 to 80 mg $m^{-2}$ orally on day 1 with intervals of 6 weeks as the first line chemotherapy. Three (5%) patients had complete and 6 (11%) partial response, and 7 (13%) patients had stable disease at least for 3 months. The patients with an objective response ($n = 9$) survived longer than the rest of the patients if the length of survival was calculated from the start of chemotherapy ($P = 0.0006$). However, the responding patients also had longer time interval from the diagnosis to the detection of distant metastases ($P = 0.05$), and survival time from disease progression following DTIC and CCNU therapy ($P = 0.005$). These findings suggest that patients with an objective response to DTIC-CCNU therapy have melanoma with a slow progression rate, and prolonged survival in such patients may in part result from the less aggressive biological nature of their tumours.

Combination chemotherapy has in nonrandomised series been reported to increase survival of the responding patients with metastatic melanoma (Seigler *et al.*, 1980), and based on such results combination chemotherapy has been recommended instead of single-agent therapy despite its greater toxicity in this uniformly fatal disease (Young *et al.*, 1985). Subcutaneous, lymph node, and pulmonary melanoma metastases respond to chemotherapy more often than visceral or osseous ones (Joensuu *et al.*, 1986; Mulder *et al.*, 1989; Thatcher *et al.*, 1989). However, disseminated melanoma with subcutaneous, lymph node, or pulmonary metastases may be associated with less aggressive biological behaviour, and, therefore, the longer survival of the responding patients might not merely result from a favourable response to chemotherapy. However, there are currently little data available in the literature indicating that responding melanoma metastases have a slower progression rate than those that do not respond to chemotherapy.

In the present retrospective series of 55 patients treated with the combination of dacarbazine (DTIC) and lomustine (CCNU) the progression rate of melanoma was assessed by calculating the time from the diagnosis to the detection of distant metastases, and the length of survival after disease progression following DTIC-CCNU therapy to death, and these estimates of rate of disease progression were correlated with response to chemotherapy.

### Materials and methods

Sixty patients with histologically diagnosed malignant melanoma with distant metastases were treated with DTIC 400 mg i.v. on days 1 to 3 and with CCNU 50–80 mg $m^{-2}$ p.o. on day 1 with 6 weeks' intervals in the Department of Radiotherapy and Oncology, Turku University Central Hospital, in 1979 to 1987. Dosage was reduced at some stage of treatment because of side-effects only in nine cases (in three responders and in six nonresponders). Fifty-five patients were evaluable and five nonevaluable for response (two patients refused to continue therapy after one course, one was lost to follow-up, in one case metastases were removed by surgery, and one patient was treated for pulmonary nodules that later turned out to be probably benign). Thirty were male and 25 female, the mean age was 52 years, range, from 21 years to 72 years. The Karnofsky's status ranged from 50 to 100, mean 83.

Dissemination of melanoma was confirmed by histology, cytology, or by radiography. Nine patients had metastases confined to subcutaneous tissue or lymph nodes only, 20 had pulmonary metastases only, 4 liver metastases only, and the remaining 22 patients had metastases in more than one location.

Previous chemotherapy had not been given, but 18 patients had been irradiated to small local fields, 40 to 60 Gy, and 12 had received adjuvant immunostimulatory treatment after the diagnosis before starting chemotherapy (three responders and nine nonresponders to DTIC-CCNU combination). Nineteen patients were treated with tamoxifen and/or various chemotherapy protocols after progression with DTIC and CCNU (five responders and 14 nonresponders).

Treatment response was assessed according to Miller *et al.* (1981). Patients with CR or PR were considered objective responders. The duration of response was calculated from the date of occurrence of remission until disease progression.

Survival analyses were done using a BMDP computer program (BMDP Statistical Software, Department of Biomathematics, University of California Press, Los Angeles, CA). Survival was estimated with the product-limit method, and comparison of survival between groups was done with the generalised Wilcoxon test (BMDP 1L). Frequency tables were analyzed using Fisher's exact test, and age distributions were compared with Student's *t*-test. All *P*-values are 2-tailed.

### Results

The mean number of cycles given was 4.1 (range, from one to 18). At writing three patients are alive with disease, and 52 (95%) have died from melanoma. Survival rate 2 years after starting chemotherapy was 15%, and the median survival time was 7 months.

The objective response rate was 16% (9/55, 3 CRs, 6 PRs), and seven patients (13%) had stable disease at least for 3 months. The duration of complete responses were 2, 2, and 18 months, and partial responses 2, 3, 3, 7, 26, and 12+ months. Objective responses were seen more frequently in patients with disease confined to subcutaneous tissue or lymph nodes (4/9, 44%), and in patients with pulmonary metastases only (4/20, 20%) than in patients with metastases elsewhere or with multiple involved sites (1/26, 4%, $P = 0.03$, Table I). All responders had Karnofsky's status 80 or more, none of the 11 patients who scored 70 ($n = 5$), 60 ($n = 5$) or 50 ($n = 1$) responded ($P = 0.18$). The mean age of the patients with objective response was 60.7 years (SD, 11.1 years), and that of the rest of the patients 50.6 years (SD, 13.9 years, $P = 0.02$). Three of the responding patients were male ($P = 0.27$).

Received 24 May 1990; and in revised form 29 August 1990.

**Table I** Response by site

| *Location of metastases* | *CR* | *PR* | *SD* | *PD* | *CR + PR/Total* |
|---|---|---|---|---|---|
| Subcutaneous or nodes only | 3 | 1 | 2 | 3 | 4/ 9 (44%) |
| Pulmonary only | 0 | 4 | 4 | 12 | 4/20 (20%) |
| Other visceral/osseous | 0 | 1 | 1 | 24 | 1/26 (4%) |
| Total | 3 (5%) | 6 (11%) | 7 (13%) | 39 (71%) | 9/55 (16%) |

The patients with an objective response lived significantly longer than those without ($P = 0.0006$, Figure 1b). The median survival time of the responders was 25 months and that of the nonresponders only 5 months. However, the responding patients had also longer a time from the diagnosis to the detection of distant metastases ($P = 0.05$, Figure 1a), and survival time from disease progression following DTIC and CCNU therapy ($P = 0.005$, Figure 1c), which suggests that patients with an objective response had melanoma with a slow progression rate. If patients with stabile disease were combined together with the responding patients in these survival analyses, the corresponding $P$ values were $P < 0.0001$ for survival after starting chemotherapy, $P = 0.09$ for distant recurrence-free survival, and $P < 0.0001$ for post-chemotherapy survival.

### Discussion

In accordance with the present results several authors have reported that patients with an objective response live a few months longer than those without a response (Seigler *et al.*, 1980; Young *et al.*, 1985; York & Foltz, 1988). Hence, chemotherapy has been thought to prolong survival of the responding patients (Seigler *et al.*, 1980; Young *et al.*, 1985), and there has been considered to be little justification for the choice of nontreatment or even single-agent chemotherapeutic regimen in advanced melanoma (Young *et al.*, 1985). However, the possibility of the responding patients to have a biologically less aggressive disease has not been taken into account when such treatment policy has been recommended. Because the responding patients had a longer time interval from the diagnosis to the appearance of distant metastases, it is likely that some of the apparent survival benefit shown in Figure 1b resulted from the slower metastatic rate and possibly from slower growth rate of the tumours of the responding patients. This hypothesis is supported by the longer time interval between disease progression after DTIC-CCNU chemotherapy and death in responding patients as compared with the nonresponding ones (Figure 1c).

DTIC is one of the most active single drugs in metastatic melanoma with a response rate of 15 to 25% (Comis, 1976; Legha, 1989). The objective response rate of 16% found in the present series is similar as we found earlier with 36 evaluable patients (Joensuu *et al.*, 1986), and also not different from the response rates obtained with DTIC alone in metastatic melanoma. Hence, the addition of oral CCNU to dacarbazine does not appear to be of much clinical value.

Higher response rates up to 40 to 45% have been reported with 3- or 4-drug regimens, such as BOLD (bleomycin, vincristine, lomustine, and DTIC, Seigler *et al.*, 1980), BELD (vincristine substituted by vindesine, Young *et al.*, 1985), and cisplatin, vindesine, and DTIC combination (Pectasides *et al.*, 1989). However, only five (25%) of the 20 evaluable patients of Young *et al.* (1985) and three (11%) of the 27 patients of Pectasides *et al.* (1989) had other visceral than pulmonary metastases, or osseous metastases. Consequently, when the BOLD regimen has been tested among patients with a larger proportion of visceral or osseous metastases, the response rate has dropped from four to 20% (The Prudente Foundation Melanoma Study Group, 1989; York & Foltz, 1988). The median survival time with BOLD has varied from 4 to 7 months (Seigler *et al.*, 1980; The Prudente Foundation Melanoma Study Group, 1989, Lakhani *et al.*, 1990), which is not superior to the median surival time of 7 months obtained

**Figure 1** **a**, Survival of the patients with malignant melanoma and with an objective response to DTIC-CCNU chemotherapy (CR/PR, $n = 9$), and the patients without an objective response (SD/PD, $n = 42$) as calculated from the data of the diagnosis to the date of detection of distant metastases. In four cases the date of excision of the primary tumour was not known with certainty or the primary tumour was not found. **b**, Survival as calculated from the start of chemotherapy to death ($n = 52$) or the last day of follow-up ($n = 3$). In CR/PR $n = 9$, in SD/PD $n = 46$. **c**, Survival as calculated from the date of disease progression following DTIC-CCNU chemotherapy to death or the last date of follow-up. In CR/PR $n = 8$ (1 patient has not had disease progression, in SD/PD $n = 46$.

in the present series, but the toxicity of the 4-drug regimen appears to be greater (York & Foltz, 1988).

There is currently no solid evidence that chemotherapy prolongs survival in disseminated melanoma, even if given as adjuvant therapy. Interferons and interleukin-2 induce tumour regression in 15 to 20% of patients, but are associated with considerable side effects (Legha, 1989). Currently, the results of chemotherapy in the treatment of disseminated malignant melanoma continue to be mostly disappointing (Ahman *et al.*, 1989). High dose chemotherapy with autologous bone marrow rescue has produced impressive response rates up to 81% with a 25% complete response rate, but the median survival value remained as 6 months with no survival benefit (Thatcher *et al.*, 1989; Lakhani *et al.*, 1990).

The present results indicate that prolonged survival of patients responding to chemotherapy as compared to those not responding to it may be poor evidence for survival benefit produced by chemotherapy. Responding tumou may have different inherent biological characteristics from the nonresponding tumours, and the alleged survival benefits based on chemotherapy response have also been criticised from a statistical point of view (Anderson *et al.*, 1983). Treatment recommendations based on such evidence should be viewed with suspicion, because treatment with toxic multidrug combinations may lead to increased costs and undue iatrogenic suffering for these fatally ill patients.

### References

AHMANN, D.L., CREAGAN, E.T., HAHN, R.G., EDMONSON, J.H., BISEL, H.F. & SCHAID, D.J. (1989). Complete responses and long-term survivals after systemic chemotherapy for patients with advanced malignant melanoma. *Cancer*, **63**, 224.

ANDERSON, J.R., CAIN, K.C. & GELBER, R.D. (1983). Analysis of survival by tumor response. *J. Clin. Oncol.*, **1**, 710.

COMIS, R.L. (1976). DTIC (NSC-45388) in malignant melanoma: A perspective. *Cancer Treat. Rep.*, **60**, 165.

JOENSUU, H., ASOLA, R. & MINN, H. (1986). Combination chemotherapy with dacarbazine and lomustine in disseminated malignant melanoma. *Acta Radiol. Oncol.*, **25**, 177.

LAKHANI, S., SELBY, P., BLISS, J.M., PERREN, T.J., GORE, M.E. & MCELWAIN, T.J. (1990). Chemotherapy for malignant melanoma: Combinations and high doses produce more responses without survival benefit. *Br. J. Cancer*, **61**, 330.

LEGHA, S.S. (1989). Current therapy for malignant melanoma. *Semin. Oncol.*, **16**, 34.

MILLER, A.B., HOOGSTRATEN, B., STAQUET, M. & WINKLER, A. (1981). Reporting results of cancer treatment. *Cancer*, **47**, 207.

MULDER, N.H., SLEIJFER, D.Th., DE VRIES, E.G.E. & 4 others (1989). Phase II study of bleomycin, dacarbazine (DTIC) and vindesine in disseminated malignant melanoma. *J. Cancer Res. Clin. Oncol.*, **115**, 93.

PECTASIDES, D., YIANNIOTIS, H., ALEVIZAKOS, N. & 5 others (1989). Treatment of metastatic malignant melanoma with dacarbazine, vindesine and cisplatin. *Br. J. Cancer*, **60**, 627.

THE PRUDENTE FOUNDATION MELANOMA STUDY GROUP (1989). Chemotherapy of disseminated malignant melanoma with bleomycin, vincristine, CCNU, and DTIC (BOLD regimen). *Cancer*, **63**, 1676.

SEIGLER, H.F., LUCAS, V.S. Jr., PHARM, B.S., PICKETT, N.J. & HUANG, A.T. (1980). DTIC, CCNU, bleomycin and vincristine (BOLD) in metastatic melanoma. *Cancer*, **46**, 2346.

THATCHER, N., LIND, M., MORGENSTERN, G. & 4 others (1989). High-dose, double alkylating agent chemotherapy with DTIC, melphalan, or ifosfamide and marrow rescue for metastatic malignant melanoma. *Cancer*, **63**, 1296.

YORK, R.M. & FOLTZ, A.T. (1988). Bleomycin, vincristine, lomustine, and DTIC chemotherapy for metastatic melanoma. *Cancer*, **61**, 2183.

YOUNG, D.W., LEVER, R.S., ENGLISH, J.S.C. & MACKIE, R.M. (1985). The use of BELD combination chemotherapy (Bleomycin, Vindesine, CCNU, and DTIC) in advanced malignant melanoma. *Cancer*, **55**, 1879.